# Pre- and postsynaptic alterations in the visual cortex of the P23H-1 retinal degeneration rat model

**DOI:** 10.3389/fnana.2022.1000085

**Published:** 2022-10-13

**Authors:** Juan R. Martinez-Galan, Maria Garcia-Belando, Jose J. Cabanes-Sanchis, Elena Caminos

**Affiliations:** Facultad de Medicina, Instituto de Investigación en Discapacidades Neurológicas, Universidad de Castilla-La Mancha, Albacete, Spain

**Keywords:** visual cortex, VGLUT, pyramidal neurons, dendritic spines, retinitis pigmentosa

## Abstract

P23H rats express a variant of rhodopsin with a mutation that leads to loss of visual function with similar properties as human autosomal dominant retinitis pigmentosa (RP). The advances made in different therapeutic strategies to recover visual system functionality reveal the need to know whether progressive retina degeneration affects the visual cortex structure. Here we are interested in detecting cortical alterations in young rats with moderate retinal degeneration, and in adulthood when degeneration is severer. For this purpose, we studied the synaptic architecture of the primary visual cortex (V1) by analyzing a series of pre- and postsynaptic elements related to excitatory glutamatergic transmission. Visual cortices from control Sprague Dawley (SD) and P23H rats at postnatal days 30 (P30) and P230 were used to evaluate the distribution of vesicular glutamate transporters VGLUT1 and VGLUT2 by immunofluorescence, and to analyze the expression of postsynaptic density protein-95 (PSD-95) by Western blot. The amount and dendritic spine distribution along the apical shafts of the layer V pyramidal neurons, stained by the Golgi-Cox method, were also studied. We observed that at P30, RP does not significantly affect any of the studied markers and structures, which suggests in young P23H rats that visual cortex connectivity seems preserved. However, in adult rats, although VGLUT1 immunoreactivity and PSD-95 expression were similar between both groups, a narrower and stronger VGLUT2-immunoreactive band in layer IV was observed in the P23H rats. Furthermore, RP significantly decreased the density of dendritic spines and altered their distribution along the apical shafts of pyramidal neurons, which remained in a more immature state compared to the P230 SD rats. Our results indicate that the most notable changes in the visual cortex structure take place after a prolonged retinal degeneration period that affected the presynaptic thalamocortical VGLUT2-immunoreactive terminals and postsynaptic dendritic spines from layer V pyramidal cells. Although plasticity is more limited at these ages, future studies will determine how reversible these changes are and to what extent they can affect the visual system’s functionality.

## Introduction

The P23H mutant rhodopsin transgenic rat is an experimental model of retinal degeneration that exhibits progressive photoreceptor loss with similar properties as human autosomal dominant retinitis pigmentosa (RP) (Berson et al., 1991; Lewin et al., 1998). Numerous studies have widely elucidated these animals’ morphology, function and molecular retina features (Machida et al., 2000; Cuenca et al., 2004; García-Ayuso et al., 2010, 2013; Kolomiets et al., 2010; Lu et al., 2013; Caminos et al., 2015; Orhan et al., 2015; Fernández-Sánchez et al., 2018). This characterization is essential to contribute to the progress of gene therapies, retina transplantation and alternative therapeutic approaches to slow photoreceptor degeneration (Aleman et al., 2001; Green et al., 2001; Zhang et al., 2003; Salzmann et al., 2006; Gorbatyuk et al., 2010; Jensen and Rizzo, 2011; Fernández-Sánchez et al., 2012; Lu et al., 2013; Rahmani et al., 2013). There are three P23H mutant rat lines with different photoreceptor degeneration rates (LaVail et al., 2018). In this study, we used only Line 1. Line 1 animals have a higher transgene expression level than Line 3 animals, and faster degeneration than the rats of Lines 2 and 3. They are also homozygous animals with a faster degeneration rate than in the heterozygote strain.

Interestingly, while the retina of these animals has been well-studied, the rest of the central nervous systems remains poorly explored. The numerous advances and the emergence of novel techniques for repairing the damaged retina in RP (Yu et al., 2017; Unal Eroglu et al., 2018), reveal the need to know whether RP affects the visual cortex structure because, although a future therapeutic strategy could partly restore the damaged retina, it might not have an effect on functional vision recovery. Electrophysical studies in the visual cortices of P23H rats have shown an increase in the spontaneous firing rate of visual cortical neurons (Wang et al., 2016), and a decrease in the firing rate, amount of information and efficiency in response to visual stimulus compared to wild-type rats (Wang et al., 2018). These findings indicate that loss of visual experience, produced by retinal degeneration, can influence cortical neuron physiology, and suggest the existence of possible morphological and/or molecular changes. However, such changes have not yet been described. In any case, the pathways that connect ganglion cells to visual cortex are relatively preserved because cortical potentials evoked by electrical stimulation of the retina in P23H rats do not decrease compared to the control rats (Koo and Weiland, 2022). In other model of photoreceptor degeneration, the rd10 mouse, the decrease in cortical activities upon visual stimulation also correlates with retinal degeneration progression (Narcisse et al., 2021). Transitory changes in early stages of RP in some vesicular neurotransmitter transporters have been described together with a net shift toward increased intracortical inhibition (Pietra et al., 2021). Anyway visual cortical circuits seems to preserve the capability of input-dependent remodeling (Begenisic et al., 2020).

There is abundant literature on the role of visual experience in maintaining central visual system integrity (Hooks and Chen, 2020). Pioneer studies on the mammalian visual cortex have shown that thalamocortical connections can be affected by loss of sensory experience. In monkeys, monocular deprivation (MD) provokes a reduction in the layer IV ocular dominance columns that correspond to the closed eye when deprivation occurs before the end of the critical period (LeVay et al., 1980). It has been also shown in kittens that the geniculocortical arbors from the deprived eye suffer a dramatic decrease in the extent and the number of branches (Antonini and Stryker, 1993). In rodents, MD alters synaptic transmission and cortical neurons cease to respond to the deprived eye stimulation via the long-term depression (LTD) mechanism (Heynen et al., 2003; Crozier et al., 2007). Therefore in P23H rats, it would be plausible to find neuroanatomical and molecular alterations due to progressive lack of visual activity owing to retinal degeneration.

In the present study, we used young postnatal P23H rats to first analyze the visual cortex structure when RP progression affected only part of the outer nuclear layer (ONL), and adult rats in the stage in which the photoreceptor layer had almost disappeared from the retina. The synaptic cytoarchitecture of the visual cortex in the P23H rats was evaluated by analyzing a series of pre- and postsynaptic parameters. At the presynaptic site, we studied the intensity of immunostaining of vesicular glutamate transporters (VGLUTs). VGLUTs are responsible for transporting glutamate to the synaptic vesicle. There are three types of VGLUTS: VGLUT1-3 (for a review, see Fremeau et al., 2004). In the cortex, VGLUT1 is present at all the layers, VGLUT2 is more specific of the thalamocortical synapsis and abounds more at layer IV (Fremeau et al., 2001; Varoqui et al., 2002; Nahmani and Erisir, 2005). In contrast, VGLUT3 is expressed by the cells that have been traditionally considered to release a different classic transmitter with a somatodendritic and axonal expression (Fremeau et al., 2002). Only scattered populations of VGLUT3 expressing neurons are found in the cerebral cortex (Herzog et al., 2004). Therefore in this study, we explored the effect of RP on VGLUT1 and VGLUT2 expressions by discarding VGLUT3 to analyze whether an imbalance exists in general excitatory neurotransmission via VGLUT1, or to specifically detect possible alterations in glutamatergic thalamic terminals, mostly VGLUT2-immunoreactive (VGLUT2-ir), from the dorsal lateral geniculate nucleus (dLGN).

Moreover at the postsynaptic site, we studied the expression of postsynaptic density protein-95 (PSD-95). PSD-95 is an important regulator of synaptic maturation by stabilizing and trafficking N-methyl-D-aspartic acid receptors (NMDARs) and α-amino-3-hydroxy-5-methyl-4-isoxazoleproprionic acid receptors (AMPARs) to the postsynaptic membrane (for a review, see Coley and Gao, 2018). We also studied spine density and distribution in cortical neurons, namely along the apical shafts of layer V pyramidal cells. These neurons are one of the main integrators of the cortical column, project to other subcortical structures and possess dendrites that span all cortical layers. Moreover, the spine density in these neurons has been demonstrated to be sensitive to visual deprivation (Ruiz-Marcos et al., 1979; Heumann and Rabinowicz, 1982; Macharadze et al., 2019). Interpreting these data together can help us to understand the visual cortex status in different RP phases.

## Materials and methods

### Animals

Transgenic P23H-1 homozygous albino (P23H) rats were kindly provided by Dr. Matthew LaVail (UCSF School of Medicine, Beckman Vision Center, San Francisco, CA, USA), and were bred in a colony at the Animal House of Universidad de Castilla-La Mancha (UCLM, Albacete, Spain). The homozygote transgenic rat line P23H-1 has an autosomal dominant rhodopsin mutation with single amino acid substitution at codon 23 (a proline to histidine substitution). These animals are on the albino Sprague Dawley (SD) background (LaVail et al., 2018). SD rats (Charles River, Barcelona, Spain and the Animal House of the Universidad de Castilla-La Mancha) were used as the wild-type controls. All the procedures were approved by institutional university committees and conformed to European and Spanish laws (Directive 2010/63/UE amended by Regulation (EU) 2019/1010, and RD 53/2013).

### Brain size quantification and cortical thickness analysis

To evaluate differences in the size of the brains, brain weight, as an estimate of the brain size, was measured in perfused brains that were subsequently used for immunofluorescence at a minimum of five animals per experimental group. For this aim, the hindbrain and olfactory bulb was previously removed. Analysis of visual cortex thickness was focused on Golgi sections by using a 4x objective together a millimeter ruler placed on the eyepiece. Measures were taken in the most medial part of monocular V1 at an approximated rostrocaudal level equivalent to Bregma –7.04 to –7.64 mm (Paxinos and Watson, 1998) at a minimum of 24 sections from 4 animals per experimental group.

### Primary antibodies

For immunohistochemistry, guinea pig polyclonal anti-VGLUT1 and anti-VGLUT2 were purchased from Merck Millipore (#AB5905 and #AB2251-I, respectively, Bilerica, MA, USA). Anti-VGLUT1 and anti-VGLUT2 were produced against the peptides corresponding to the C-terminus of rat VGLUT1 and VGLUT2. Mouse monoclonal antibody anti-microtubule-associated protein 2 (MAP2) was obtained from Sigma (#M 4403, clone HM-2, St. Louis, MO, USA) and raised against rat brain MAP2.

For the Western blot, mouse monoclonal anti-PSD-95 was obtained from Merck Millipore (#MAB1596, Bilerica, MA, USA). The antibody was raised against a recombinant rat PSD-95 protein. A mouse monoclonal antibody to Glyceraldehyde-3-phosphate dehydrogenase (anti-GAPDH) from Thermo Fisher Scientific (#AM4300, clone 6C5, Madrid, Spain) was used as a load control.

### Tissue processing for immunofluorescent labeling

Twelve female rats, aged 30 and 230 postnatal days (P), were used, specifically three animals per age (P30 and P230) and strain (SD and P23H). Animals were deeply anesthetized with ketamine (100 mg/kg, Parke-Davis, Alcobendas, Spain) and xylazine (10 mg/kg, Dibapa, Barcelona, Spain). Rats were perfused through the left ventricle with 4% paraformaldehyde in 0.1 M phosphate buffer (PB), pH 7.4. Eyes and brains were dissected out and post-fixed for 4 h in fresh fixative at 4°C. Tissues were washed, transferred to PB containing 30% sucrose and embedded in Tissue Tek (Leica, Wetzlar, Germany). The retinal and neocortical coronal sections containing the primary visual cortex (V1) were obtained at 16 μm with a cryostat (Leica CM3050S) and mounted onto Super Frost slides (Kindler, Freiburg, Germany). The retina structure was evaluated in sections, which were washed in phosphate buffered saline, pH 7.3, containing 0.25% Triton X-100 (PBST), air-dried in the dark and mounted and coverslipped with Fluoroshield with DAPI (Sigma, #F6057, Madrid, Spain). Cortical sections were processed for immunofluorescent labeling as follows.

### Double immunofluorescent labeling

Anti-VGLUT1 and anti-VGLUT2 staining was combined with DAPI nuclear labeling and anti-MAP2 to identify somata and dendrites. Cryosections were pretreated with 2% BSA, 3% normal horse serum in PBST at room temperature (RT) for 30 min and then incubated with anti-VGLUT1 (1:500) or anti-VGLUT2 (1:1,000) and anti-MAP2 (1:500) at (RT) for 24 h. All the following steps were carried out in the dark at RT. After several rinses in PBST, sections were incubated for 60 min by using the following secondary antibodies: Biotinylated goat anti-guinea pig IgGs (Vector Laboratories Inc., Burlingame, CA, USA), diluted at 1:100; Cy5-conjugated goat anti-mouse IgGs (Jackson ImmunoResearch, Baltimore Pike, PA, USA), diluted at 1:200. After rinsing, sections were incubated in Alexa 488-conjugated streptavidin (Life Technologies, Gran Island, NY, USA) for 60 min, and diluted at 1:1,000. After rinsing, sections were air-dried in the dark and mounted with Fluoroshield with DAPI.

### Confocal microscopy and quantification

VGLUT1 immunolabeled coronal sections were examined in V1 under a Zeiss LSM 800 laser scanning confocal microscope equipped with excitation laser lines at 405, 488, 561, and 640 nm. Confocal image stacks were recorded through separate channels for DAPI (abs: 359 nm, em: 461 nm), Alexa 488 (abs: 498 nm, em: 520 nm) and Cy5 (Cy5 abs: 650 nm, em: 670 nm). By using the same confocal settings, no labeling was found in the control sections incubated in the absence of primary antibodies. For VGLUT2 immunolabeling, a Zeiss LSM 710 laser scanning confocal microscope of continuous spectral detection, equipped with excitation laser lines at 405, 458, 488, 514, 561, and 633 nm, was used.

To quantify VGLUT1 labeling, confocal series of images were captured at the layers II/III of V1 using a 63x oil objective (Zeiss, NA = 1.4). For VGLUT2, images were captured at layer IV employing a 40x oil objective (Zeiss, NA = 1.3), which allows to distinguish the limit of layer IV. The same confocal settings (laser intensity, pinhole, gain), which had been previously adjusted to obtain an optimal signal and contrast and to avoid the saturation of pixels, were used for each marker. Next the single images from every z-stack that had the highest mean gray value (MGV), measured by the Zeiss LSM software, were selected. All the single images had the same optical thickness (0.5 μm) and surface (26,412 and 19,380 μm^2^ for VGLUT1 and VGLUT2, respectively). Twenty-four images per experimental condition (4 images per section, 2 sections per animal, 3 animals per condition) were obtained and processed by the Fiji program (Schindelin et al., 2012), the open-source platform software based on ImageJ.^1^ Images were transformed to 8 bits and a single background value, that allowed to reach an acceptable signal-to-noise balance, was assigned to each set of experiments performed under identical experimental conditions (Supplementary Figure 1). This value was calculated after measuring MGV in non-specific stained pixels. The same range of gray values (0–255) was used for all images. While the MGV of the whole images was used as an indicator of the protein levels in a specific cortex field, the percentage of the immunostained area indicated the area occupied by the VGLUTs-ir profiles labeled in that field.

### Western blots

Animals were sacrificed by decapitation after inducing their anesthesia with 4% isoflurane (Zoetis, Madrid, Spain). Brains were immediately removed, and the visual cortex was isolated and frozen in liquid nitrogen. All the tissues were stored at –80°C until used. At least three animals per age (P30 and P230) of each rat strain (SD and P23H) were employed. Tissues were homogenized in 1,000 ml of ice-cold lysis buffer (50 mM TrisHCl, pH 7.5, 150 mM NaCl, 2 mM EDTA, 0.1% SDS, 0.5% deoxycholate, 0.2 mM Na3VO4, 50 mM NaF, 1% Triton X-100, 1 mM PMSF, 0.5 μg/ml leupeptin, 1 μg/ml aprotinin). Homogenates were centrifuged at 12,000 g for 15 min at 4°C and supernatants were stored at 20°C. The protein concentration was determined by the BCA Protein Assay kit (Thermo Fisher Scientific, Rockford, IL, USA). Forty mg of whole protein extracts were electrophoresed on 10% SDS polyacrylamide gels using the mini-PROTEAN III system (Bio-Rad, Hercules, CA, USA) for over 100 min at 120 V. Gels were transferred to nitrocellulose membranes (GE Healthcare Bio-Sciences, Uppsala, Sweden) for 30 min at 12–15 V using a semidry blotter (Bio-Rad). Immunodetection was performed by blocking the non-specific binding in the blots with Tris-buffered saline Tween-20 (TBST: 50 mM Tris, pH 7.5, 200 mM NaCl, 0.1% Tween 20 and 10% bovine serum albumin) for 1 h at RT. Then membranes were incubated with the primary monoclonal antibody anti-PSD-95 (1:1,000) in TBST- 5% BSA overnight at 4°C, and subsequently with the horseradish peroxidase-conjugated secondary goat anti-mouse IgG (Jackson ImmunoResearch) and diluted at 1:2,000 for 1 h at RT. Next membranes were developed by means of a chemiluminescence assay (ECL™ start Western blotting detection reagent, Amersham™) for 5 min and were scanned in a computer equipped with an LAS-mini 3,000 system (Fujifilm, Tokyo, Japan). Finally, membranes were washed with TBST and exposed to monoclonal anti-GAPDH (Thermo Fisher Scientific) diluted at 1:8,000 for 1 h, followed by the horseradish peroxidase-conjugated secondary anti-mouse IgG (Jackson ImmunoResearch) diluted at 1:2,000 for 1 h at RT. The controls included (1) extracts of cerebellum and (2) membranes incubated with only the secondary antibodies. Quantification was performed with Fiji. Statistical data were determined from at least three different samples in triplicate per each age point.

### Golgi-cox staining

Sixteen female rats, aged P30 and P230 were anesthetized with 4% isoflurane (flow rate of 1 L/min O_2_) inside an appropriate chamber prior to decapitation. Brains were stained using the FD Rapid GolgiStain kit (FD NeuroTechnologies, Columbia, MD, USA) following the manufacturer’s instructions. Brains were extracted, rinsed in distilled water, separated into two hemispheres for better impregnation and immersed in impregnation solution A + B in a 1:1 mixture and stored at RT for 2 weeks in the dark. This solution contained mercuric chloride, potassium dichromate and potassium chromate. It was prepared 24 h prior to use and was replaced after the first 24 h of immersion. Brains were transferred to the cryoprotection solution C containing 30% sucrose at RT in the dark for 72 h up to 1 week. Solution C was replaced after the first 24 h of immersion. Hemispheres were embedded in Tissue Tek (Leica), immersed in buckets containing isopentane and frozen in liquid nitrogen. The coronal sections (200 μm) containing V1 were obtained with a cryostat (Leica CM3050S) and transferred to gelatin-coated slides by applying a few drops of solution C. Sections were dried naturally at RT in the dark. Slides were rinsed twice in Milli-Q water for 4 min and placed for 10 min in the staining solution that contained 25% of solution D (ammonia), 25% of solution E (sodium thiosulfate) and 50% of Milli-Q water. After two rinses in Milli-Q water, sections were dehydrated in sequential rinses of 50, 75, and 95% ethanol (for 4 min each rinse) and 4 times in 100% ethanol. Sections were cleared in xylene 3 times for 4 min each rinse and coverslipped with DPX.

The complete and well-impregnated apical shafts of the layer-V pyramidal neurons in V1 were selected at low magnifications (10x and 40x). Dendritic spines were counted at the 60x magnification in each 50 μm-long segment of the selected apical shafts starting from the cell body. This was done for three neurons of each rat for four rats/experimental group so that 12 neurons were studied per experimental group. Counts were made on coded tissue samples so that the animal’s experimental condition was not known to the observer. The spine distribution along the apical shafts and the mean number of spines every 50 μm were analyzed.

### Statistical analysis

The statistical significance between the mean values of the brain weight, cortical thickness, MGV and area fraction was assessed by either the Student’s *t*-test for the parametric data or the Mann-Whitney *U*-test when data were not normally distributed by using GraphPad Prism 5.0 (GraphPad Software, La Jolla, CA, USA). For Western blots, statistical significance was analyzed by a Mann-Whitney *U*-test. To assess the significance of the differences in the spine distribution between the SD and P23H rats, the two-way analysis of variance (ANOVA) was carried out. The factors involved in this study were the experimental condition (RP) and the distance to the cell body. The statistical significance among the mean number of spines every 50 μm corresponding to two different groups was assessed by a Mann-Whitney *U*-test. Throughout the text, values are expressed as arithmetic mean ± standard error of the mean (SEM).

## Results

### Retinal and brain phenotypes in the P23H rats

**FIGURE 1:**
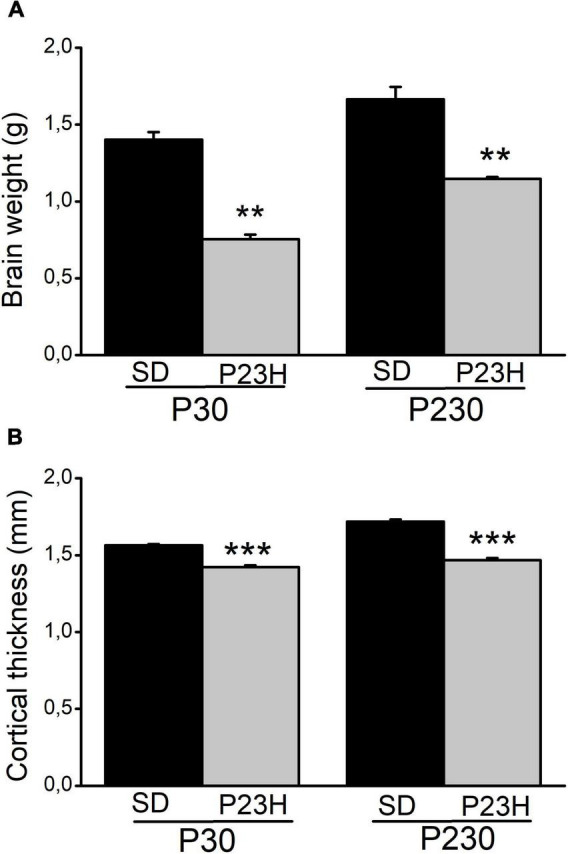
Brain weight and visual cortical thickness of the SD and P23H rats at P30 and P230. **(A)** Brain weight and **(B)** cortical thickness increased with age in both conditions but they were smaller in P23H compared to the SD rats. Differences were statistically significant with **p < 0.01 according to the Student’s *t*-test **(A)** and with ***p < 0.001 according to the Mann-Whitney *U*-test **(B)**.

The retina sections from the SD and P23H rats were analyzed to evaluate the retina structure in early (P30) and late (P230) degeneration stages by DAPI nuclear labeling (Supplementary Figure 2). During early retinal degeneration, at P30, ONL thickness dramatically decreased compared to the normal SD retinas. Although the whole retina thickness decreased, inner layers apparently remained unaltered. Later at P230, the ONL almost completely disappeared. Subsequently, a more noticeable decrease in retina thickness was observed. We also observed a reduction in the body size of all the analyzed P23H rats compared to the SD rats of the same age (data not shown). Moreover, the brain size of the P23H rats also decreased at both ages vs. the SD rats (Supplementary Figure 3). To evaluate this difference, we measured the brain weight, as an estimate of the brain size (Figure 1A). In the P23H rats, at both the studied ages, brain weight significantly decreased with *p* < 0.01 (Mann-Whitney *U*-test) compared to the SD rats. The mean values (± SEM) of the brain weights for the P23H rats at P30 and P230 were 53.81 ± 2.64% and 68.99 ± 0.90%, respectively, from those found in SD. Then we determined the visual cortex thickness in the SD and P23H rats in the Golgi sections (Figure 1B). The cortical thickness in the P23H rats at both the studied ages significantly decreased with *p* < 0.001 (Student’s *t*-test) compared to the SD rats. The mean values (± SEM) of the cortical thickness for the P23H rats at P30 and P230 were 90.91 ± 0.19% and 85.39 ± 0.83%, respectively, from those found in SD. This was not a specific effect of retinal degeneration on V1, because all the neocortex was apparently thinner in the P23H rats.

### Vesicular glutamate transporter 1 distribution in the cortices of the young and adult Sprague Dawley and P23H rats

VGLUT1 is a key molecule in transporting glutamate to the synaptic vesicles widely expressed in the excitatory synapsis from all the cortical layers. To study a possible imbalance in the excitatory visual cortical circuitry due to RP, we analyzed the pattern of VGLUT1 immunolabeling in V1 at P30 and P230 by merging VGLUT1 with DAPI and MAP2 immunolabeling to both facilitate orientation through the different layers.

**FIGURE 2:**
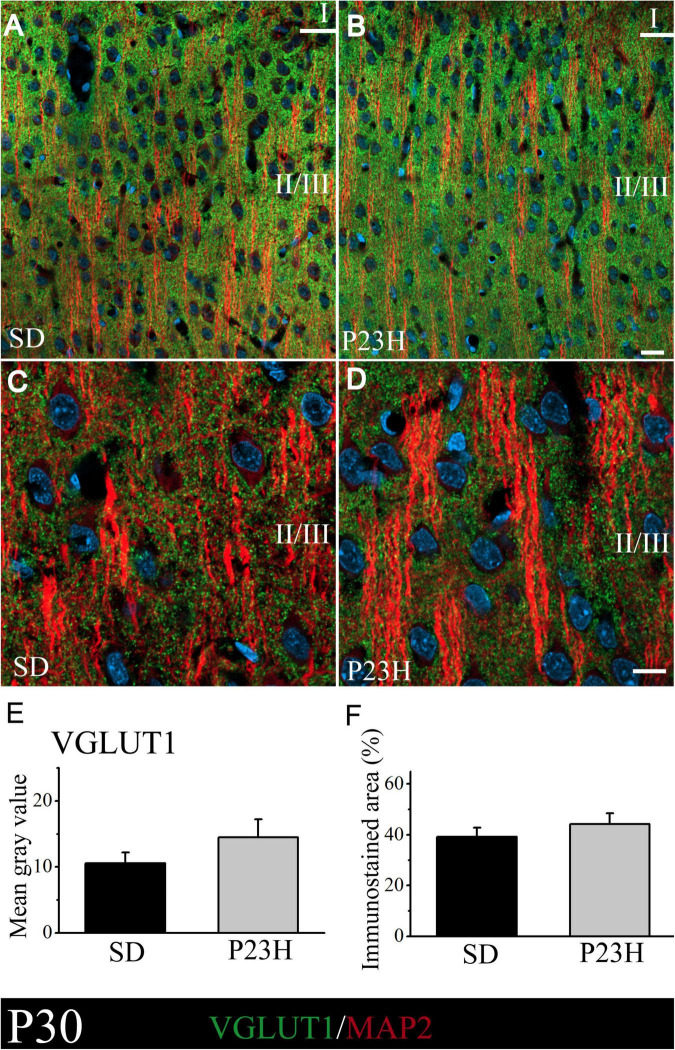
VGLUT1 and MAP2 immunolabeling in the visual cortices of the SD and P23H rats at P30. **(A,B)** Confocal images of the VGLUT1-ir presynaptic structures (green) and MAP2-ir somata and dendrites (red) along the supragranular layers of the visual cortices of the young SD and P23H rats. Nuclei are counterstained with DAPI (blue). **(C,D)** High-magnification confocal images of VGLUT1 and MAP2 immunolabeling of visual cortical layers II/III. VGLUT1-ir terminals are homogeneously distributed and the amount of VGLUT1 seems similar for both conditions. **(E,F)** Quantification of the MGV and % of immunostained areas. No statistically significant differences were found. Scale bar: 20 μm.

At P30, VGLUT1 immunolabeling was homogeneously distributed through all the cortex layers. A low-magnification image of the upper visual cortex layers of the SD and P23H rats at P30 is shown in Figures 2A,B, where the MAP2-ir apical shafts of the pyramidal neurons, which ascend to layer I, are visualized among the VGLUT-ir boutons. Higher magnifications of layer II/III showed no apparent differences in the VGLUT1 staining between both conditions (Figures 2C,D). The quantification of VGLUT1 immunofluorescence by determining the MGV and stained area at layers II/III confirmed this observation. No significant differences were detected in any of the studied parameters (Figures 2E,F). The MGV (± SEM) obtained from the sections of the SD and P23H animals were 10.52 ± 1.63 and 14.47 ± 2.73, respectively (Figure 2E). The mean values (± SEM) of the percentage of the immunoreactive area from the SD and P23H rats were 39.13 ± 3.62% and 44.25 ± 4.16%, respectively (Figure 2F).

**FIGURE 3:**
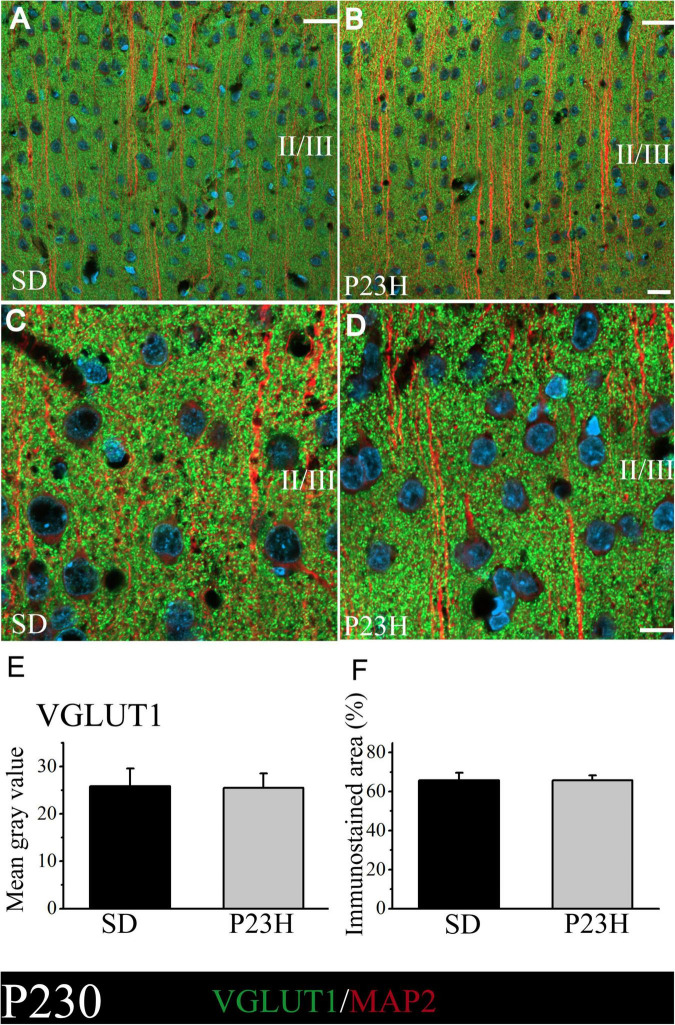
VGLUT1 and MAP2 immunolabeling at P230 in the visual cortices of the SD and P23H rats at P230. **(A,B)** Confocal images of the VGLUT1-ir presynaptic structures (green) and MAP2-ir somata and dendrites (red) along the supragranular layers of the visual cortices of the adult SD and P23H rats. Nuclei are counterstained with DAPI (blue). **(C,D)** High-magnification confocal images of VGLUT1 and MAP2 immunolabeling of visual cortical layers II/III. The same pattern of VGLUT1 immunolabeling is found for both conditions. **(E,F)** Quantification of the MGV and % of immunostained areas. No statistically significant differences were found between both conditions. Scale bar: 20 μm.

At P230, we apparently found an increase in immunolabeling compared to the P30 rats. The intensity of labeling was similar between the SD and P23H rats at P230, as shown by the low- and higher-magnification images of the upper visual cortex layers (Figures 3A–D). This observation was confirmed by the VGLUT1 immunofluorescence quantification, where no significant differences between groups were found. The MGV obtained from the sections of the SD and P23H animals were 25.84 ± 3.70 and 25.48 ± 3.01, respectively (Figure 3E). The percentage of the immunoreactive area from the SD and P23H rats were 65.82 ± 3.85% and 64.74 ± 2.59%, respectively (Figure 3F). The results obtained at P30 and P230 indicated that the visual deficits detected in the P23H rats did not seem to affect the VGLUT1 distribution at either of the two studied ages.

### Vesicular glutamate transporter 2 distribution in the cortices of the young and adult Sprague Dawley and P23H rats

**FIGURE 4:**
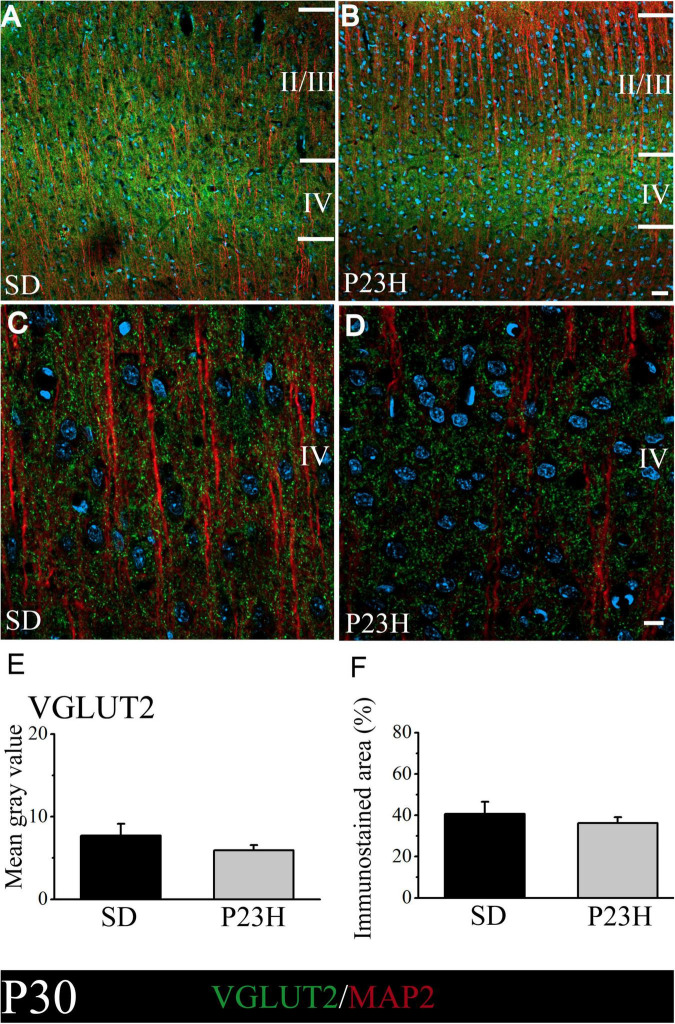
VGLUT2 and MAP2 immunolabeling in the visual cortices of the SD and P23H rats at P30. **(A,B)** Confocal images of the VGLUT2-ir thalamic presynaptic structures (green) and MAP2-ir somata and dendrites (red) along layers II/III and IV of the visual cortices of the young SD and P23H rats. Nuclei are counterstained with DAPI (blue). Note a well-defined and narrower VGLUT2-ir band in P23H. **(C,D)** High-magnification confocal images of VGLUT2 and MAP2 immunolabeling of visual cortical layer IV. **(E,F)** Quantification of the MGV and % of immunostained areas. No statistically significant differences were found between both conditions. Scale bar: 25 μm **(A,B)**; 10 μm **(C,D)**.

We then studied whether VGLUT2, a vesicular glutamate transporter that predominates in the thalamic afferences, was altered in the P23H rats. The objective was to elucidate if in early or late stages RP could alter the pattern of excitatory afferences that arrive from dLGN to layer IV. At P30, the low-magnification pictures depict a diffuse VGLUT2 band at layer IV, which moderately extended to upper layers. The VGLUT2 band seems more evident, but narrower, and is restrained to layer IV in the P23H rats (Figures 4A,B). The higher magnifications pictures of layer IV show no evident differences in the VGLUT2-ir labeling between the SD and P23H rats (Figures 4C,D). The MGV of the SD and P23H rats were 7.68 ± 1.44 and 5.94 ± 0.60, respectively. The percentage of the immunoreactive area was 40.63 ± 5.84% for the SD and 36.23 ± 3.81% for the P23H rats. Differences were not statistically significant (Figures 4E,F).

**FIGURE 5:**
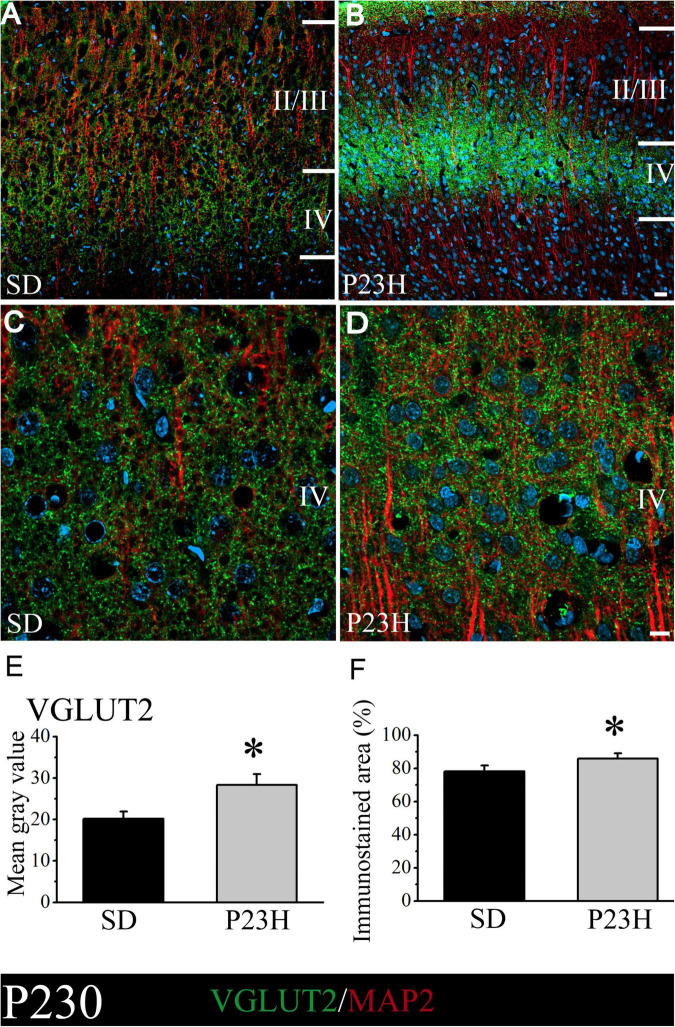
VGLUT2 and MAP2 immunolabeling in the visual cortices of the SD and P23H rats at P230. Nuclei are counterstained with DAPI (blue). **(A,B)** Confocal images of the VGLUT2-ir thalamic presynaptic structures (green) and MAP2-ir somata and dendrites (red) along layers II/III and IV of the visual cortices of the adult SD and P23H rats. Layer IV VGLUT2-ir band in P23H was stronger and narrower compared to the SD rats. **(C,D)** High-magnification confocal images of the VGLUT2 and MAP2 immunolabeling of visual cortical layer IV. **(E,F)** Quantification of the MGV and % of immunostained areas. In both cases, differences were statistically significant with *p < 0.05 according to the Student’s *t*-test **(E)** and the Mann-Whitney *U*-test **(F)**. Scale bar: 25 μm **(A,B)**; 10 μm **(C,D)**.

At P230, the low-magnification images also show that the layer IV immunoreactive band in P23H is apparently much narrower, but stronger, compared to the SD rats (Figures 5A,B). This was confirmed in the higher magnification images by the qualitative evaluation (Figures 5C,D) and by the quantitative analysis of immunofluorescence (Figures 5E,F). The SD and P23H levels of the MGV were 20.13 ± 1.78 and 28.33 ± 2.57, respectively. This difference was found to be statistically significant by the Student’s *t*-test (*p* < 0.05). The percentage of the immunoreactive area from the SD and P23H rats were 78.22 ± 3.43% and 85.82 ± 3.22%, respectively. The difference was also statistically significant according to the Mann-Whitney *U*-Test (*p* < 0.05).

### Postsynaptic density protein-95 expression in the cortices of the young and adult Sprague Dawley and P23H rats

**FIGURE 6:**
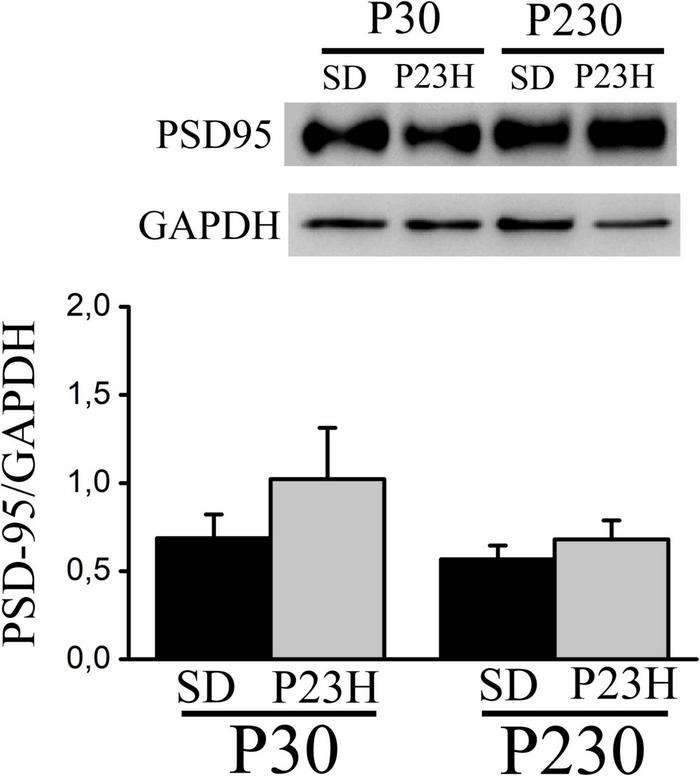
Western blot showing PSD-95 from the extracts of the visual cortices of the control SD and P23H rats at P30 and P230. The antibody recognizes a band of approximately 100 kDa. No differences among groups were found. Detection of GAPDH was included as an internal control and used to calculate the relative amount of PSD-95.

We analyzed by Western blot the PSD-95 expression in the visual cortices of the P30 and P230 rats to evaluate whether RP could affect this postsynaptic density component. In all the groups, we found one band of approximately 100 kDa. The densitometric analysis showed no differences among groups. At P30, the mean values (± SEM) of the relative amount of protein were 0.68 ± 0.13 and 1.02 ± 0.29 for the SD and P23H rats, respectively. At P230, the values were 0.56 ± 0.07 for the SD and 0.68 ± 0.10 for the P23H rats (Figure 6).

### Spine distribution along the apical shafts of the layer V pyramidal neurons in the cortices of the young and adult Sprague Dawley and P23H rats

**FIGURE 7:**
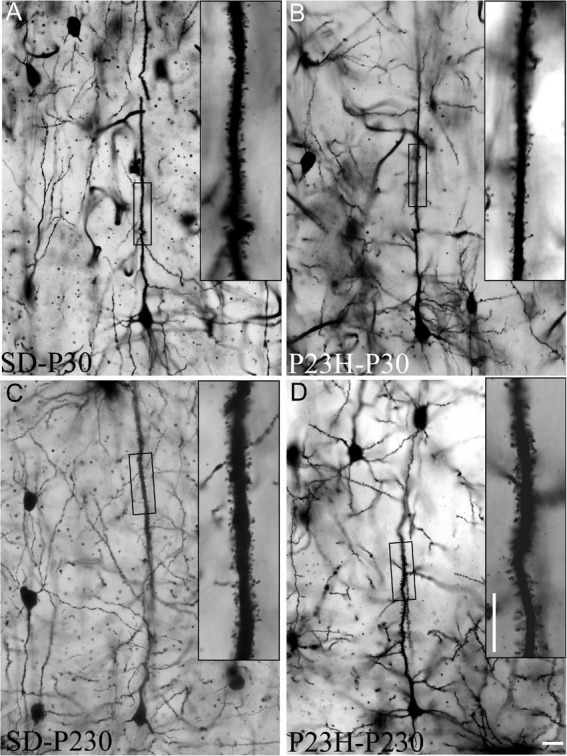
Golgi-stained sections from the primary visual cortices of the SD and P23H rats at P30 and P230. **(A–D)** The low-magnification image shows examples of the layer V pyramidal neurons selected for the quantitative evaluation of dendritic spines. Insets show dendritic spines from representative apical shaft segments. Scale bar: 10 μm.

The Golgi-Cox method was used to visualize dendritic spines, which were counted in each 50 μm-long segment of the selected apical shafts of the layer V pyramidal neurons. Figures 7A–D shows the layer V pyramidal neurons of the SD and P23H rats at P30 and P230. Spines are observed at a higher magnification of apical shafts.

**FIGURE 8:**
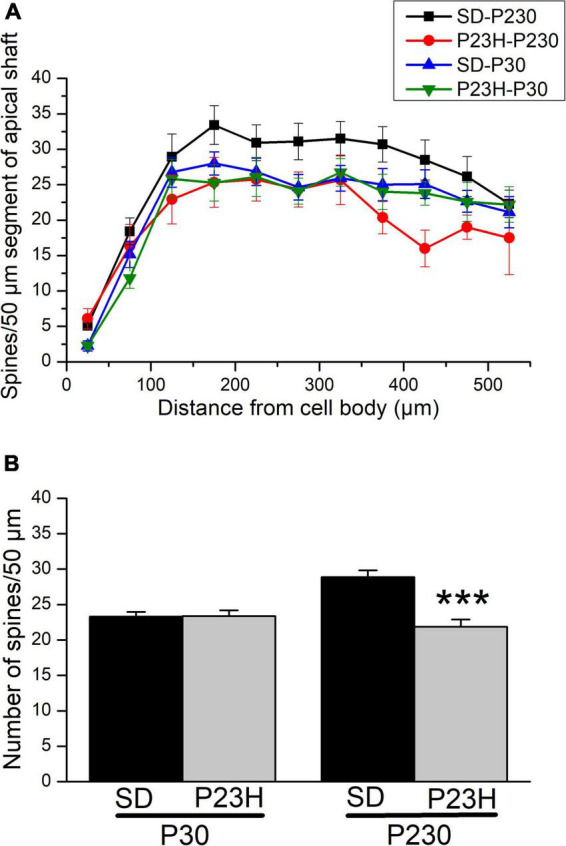
Distribution and spine density along the apical shafts of the layer V pyramidal neurons of the visual cortices of the SD and P23H rats at P30 and P230. **(A)** Dendritic spine distribution along the apical shafts. An increase in the number of spines occurs with age, but only in the control SD rats. The spine distribution in the P23H-P230 rats was closer to that observed in both groups of young P30 rats. A significant decrease in the number of spines was observed in the P23H-P230 rats vs. SD-P230. The two-way ANOVA showed at P230 that these differences were statistically significant (***p < 0.001). **(B)** Mean number of dendritic spines/50 μm in the apical shafts. At P30, no differences were found between the mean number of dendritic spines/50 μm between the SD and P23H rats. At P230, a significant decrease in the number of spines in the P23H animals is observed vs. SD (*p* < 0.001; Mann-Whitney *U*-test).

Figure 8A shows the dendritic spine distribution along the apical shafts of the SD and P23H rats at P30 and P230. As expected, this figure depicts how the number of spines in the initial segments is smaller, and increases when the count is made on the segments further from the soma. At P30, the spine distributions in both SD and P23H were similar, and the two-way ANOVA applied of the data resulted in a non-significant F value, which corresponded to the retinal degeneration effect [*F*(1, 217) = 0.83]. The spine distribution in the P23H rats at P230 reflected major spine loss in relation to SD due to a more dramatic retinal degeneration effect on the cortical circuitry at this age. The *F*-value in spìne distribution due to RP was significant with *p* < 0.001 [*F*(1, 208) = 25.01]. It is noteworthy that age came over as an important factor in the spine distribution in the normal animals. The number of spines in the adult SD P230 rats increased compared to the young SD P30 rats. The *F*-value in spine distribution due to age was significant in the SD rats with *p* < 0.001 [*F*(1, 219) = 17.19]. However, no increase in the number of spines due to age occurred in the P23H rats, where the spine distribution along apical shafts did not change. The *F*-value in spine distribution due to age was not significant in P23H rats of different ages *[F*(1, 206) = 0.24]. RP prevents neurons from late maturation and keeps them youthful.

When all the data measured in the apical shafts of the layer V pyramidal neurons from the same experimental condition were pooled, we obtained the mean number of dendritic spines every 50 μm (Figure 8B). As the initial segment contained only a few spines, the 50 μm initial segment was excluded from this quantification. The data at a distance from the soma of between 50 and 550 μm were included. At P30, no differences were found between the mean number of dendritic spines every 50 μm between the SD and P23H rats. The mean values (± SEM) obtained were 23.31 ± 0.66 and 23.39 ± 0.79, respectively. However, at P230, a significant decrease in the number of spines in the P23H animals was observed vs. SD, with *p* < 0.001 (Mann-Whitney *U*-test). The mean values at this age were 28.88 ± 0.95 for the SD and 21.86 ± 1.03 for P23H rats.

## Discussion

Here we study the effect of photoreceptor degeneration in the synaptic organization of the visual cortices of the young (P30) and adult (P230) rats. To this end, we used pre- and postsynaptic markers, which are widely described in the literature, together with an analysis of the spine distribution in the pyramidal layer V neurons from SD and P23H rat visual cortices. The major findings of this study were: (1) In the young rats, RP did not affect the expression of presynaptic markers or PSD-95; (2) although no differences in VGLUT1 distribution in layers II/III or PSD-95 expression were found between both groups in the adult rats, an altered pattern of the VGLUT2-ir thalamocortical terminals that arrive at layer IV was observed in the P23H rats at P230; (3) while RP had no effect on density and spine distribution along the apical shafts of the pyramidal neurons in the young rats, it significantly decreased spine density and altered spine distribution in the adult P23H rats.

### Phenotypic alterations of the P23H rats

The retinal degeneration pattern herein described agrees with that previously described in the P23H-1 line (Caminos et al., 2015; LaVail et al., 2018). At P30, the ONL thickness dramatically dropped and had almost disappeared at P230. The whole retina thickness also decreased from P30 to P230. However, as previously described, the number of retinal ganglion cells should not drastically drop at the studied ages compared to their homologous SD rats (García-Ayuso et al., 2010). Although these authors indicate that the number of retinal ganglion cells at P180 and P270 is significantly smaller in the P23H than the SD rats, the differences do not seem to be enough to compromise the integrity of the visual pathway from the retinal ganglion cells to the visual cortices in the P23H rats. The alterations herein described in the visual cortex at P230 might be due to the reduction in sensory activity, instead of optical nerve degradation and a reduction in the terminals arriving from the dLGN to the visual cortex.

Beyond retinal degeneration, we also found other important phenotypical changes in the P23H rats. We previously reported hearing impairment by showing auditory deficits and cochlear abnormalities (Sotoca et al., 2014). We observed an evident decrease in the body weight and brain size of the P23H rats vs. SD. Then we analyzed the cortex thickness at the level of V1 and found that it also decreased. However, neither the cortical structure nor laminar organization seemed to have altered. Although it would be very interesting to analyze these changes in depth and establish how a mutation in the rhodopsin gene can produce such marked morphological changes, this was not the objective of the present work.

### Effect of retinitis pigmentosa on the presynaptic distribution of markers in the visual cortices of the young and adult rats

We first checked the presence of VGLUT1 in the V1 from the SD and P23H rats. In the P23H rats, we wondered if loss of sensory activity could lead to a deficit of VGLUT1 in the visual cortex. It is known in certain neurodegenerative disorders that VGLUT1 expression can decrease and have devastating consequences for cognitive function (Sokolow et al., 2012; Giuliani et al., 2019). Deficit of VGLUT1 reduces glutamatergic transmission intensity and impairs visual attention, short-term plasticity (Lindström et al., 2020) and long-term potentiation (Balschun et al., 2010). In a first qualitative evaluation performed under a confocal microscope at all the cortical layers, we did not observe any apparent differences between groups. We later specifically analyzed VGLUT1 distribution by measuring the MGV and the percentage of the immunostained areas at layers II/III where the glutamatergic axons from granular cells, containing VGLUT1, come into contact with the apical dendrites from pyramidal cells. No differences were seen at either age between the SD and P23H rats, not even at P230 when lack of sensory activity was more prominent. These results suggest that the global balance of excitatory neurotransmission represented by VGLUT1 terminals is preserved in the P23H rats. VGLUT1 containing terminals at layers II/III originate most likely from different types of neurons, which include granular cell axons, collaterals from pyramidal neurons and cortico-cortical afferences. Some might not be sensitive to loss of visual activity. However, another possibility that we cannot rule out is the existence of a lower absolute number of VGLUT1 terminals at layers II/III in the P23H rats, but in a smaller volume, due to the decrease in the thickness of the P23H cortex. To observe dramatic changes in the VGLUT1 distribution, an insult before the critical period is necessary, which occurs in the hypothyroidism when this is induced experimentally in embryonic or early postnatal development (Navarro et al., 2015).

Furthermore, VGLUT2-immunoreactivity seems to depend more on sensory activity (Mainardi et al., 2010) because it is the predominant transporter upon layer IV thalamocortical synapsis (Nahmani and Erisir, 2005). In our study, the pattern of VGLUT2 distribution changed gradually with age in the P23H rats to a narrower band. In deafferented kittens, the thalamocortical terminals from the deprived eye dramatically decrease in arborization (Antonini and Stryker, 1993). It was also expected a decreased of VGLUT2 immunolabeling due to the lack of visual activity in the P23H rats. However, it is intriguing that immunolabeling of these terminals at layer IV of the P23H rats is more intense than that in the SD rats. Here also exists the possibility that VGLUT2 terminals at layer IV occupy a smaller volume in the P23H rats resulting in an increase in the intensity of immunolabeling, without a significative increase in the absolute number of terminals.

### Effect of retinitis pigmentosa on postsynaptic marker expression and spine distribution in the young and adult rats

The next step was to analyze the effect of RP on the PSD-95 expression in the visual cortex. In the mature synapsis, PSD-95 is linked with the NMDA receptor through the interaction between its PDZ domains and the NR2 subunit (Kornau et al., 1995), and also with the AMPA receptors through the interaction with stargazin (Chen et al., 2000). PSD-95 regulates the size and strength of synapses (Ehrlich and Malinow, 2004), and promotes synaptogenesis and spine formation (Nikonenko et al., 2008). Therefore, differences between experimental groups would indicate an alteration to the postsynaptic scaffold, which would be more probable in adult rats. Unlike other neurodegenerative pathologies like Alzheimer’s disease (Shao et al., 2011), RP does not bring about changes in PSD-95 expression, at least not at the studied ages. We must add the limitations of the technique, where a part of the cortical neighboring areas to the visual cortex are extracted and processed together with our region of interest. It is likely that postsynaptic differences can be very subtle and affect only certain neuronal subpopulations.

For this reason, we focused on the layer V pyramidal neurons. These neurons are one of the main integrators of the cortical column and project to other subcortical structures. Previous works have demonstrated that the number of dendritic spines from layer V pyramidal neurons is sensitive to visual deprivation (Ruiz-Marcos et al., 1979; Heumann and Rabinowicz, 1982). At P30, the spine distribution along the apical shafts between the SD and P23H rats was similar. In contrast, at P230 we observed a decrease in the number of spines along the different segments analyzed in the P23H group vs. the SD rats. Moreover, the age-related increase in spines that normally occurs after P30 (Ruiz-Marcos et al., 1992) did not occur in the P23H rats, whose spine distribution in adult P230 resembles that which occurs in juvenile stages. Furthermore, when we analyzed the mean number of spines in all the segments, and independently of the distance to the soma, we once again found a significant decrease in spine density, but only in the adult P23H rats compared to the SD animals. Despite the differences between RP and the above-described experimental models, rats were left in the dark (Ruiz-Marcos et al., 1979) or bilateral enucleation occurred in mice (Heumann and Rabinowicz, 1982), in all cases, the result was loss of dendritic spines after decreased visual activity. Although layer V pyramidal neurons provide an excellent model to study neocortical postsynaptic changes during photoreceptor degeneration, our study had some limitations, since in rodent cortex there are two subpopulations of layer V pyramidal neurons that have different dendritic morphologies, projection targets, and electrophysiological properties. Our data revealed a decrease in the number of dendritic spines in the layer V pyramidal neurons, but without analyzing the effect on a specific subpopulation. Type I pyramidal neurons have thick-tufted apical dendrites, project to subcortical structures (spinal cord, superior colliculus and pontine nucleus), fire bursts of action potentials, and their somata are located in the upper part of the layer V. Type II neurons are non-tufted pyramidal cell, project to the contralateral cortex and the ipsilateral striatum, never fire bursts of action potentials, and their somata are located in the lower part of the layer V (Larkman and Mason, 1990; Kasper et al., 1994).

In the future, it would be interesting to extend the analysis to pyramidal layer III neurons, which are also sensitive to lack of visual experience (Heumann and Rabinowicz, 1982; Macharadze et al., 2019), and to even elucidate how RP can affect learning and memory consolidation stages by studying distinct types of spines that represent different degrees of maturation, although this objective would require other experimental approaches (Risher et al., 2014; Bączyńska et al., 2021).

## Conclusion

In conclusion, in the first retinal degeneration stages (P30), the synaptic organization of V1 was not affected by RP, and was probably maintained by the activity of the still preserved retinal ganglion cells. However, later RP stages (P230) led to more noticeable changes in presynaptic (thalamocortical VGLUT2-ir terminals) and postsynaptic elements (dendritic spines from pyramidal layer V). To our knowledge, the present study shows morphological changes in the visual cortices of rodents in a model of RP for the first time. It can be useful to establish the relation among the morphological, physiological and molecular events that occur in the visual cortex during RP or in other models of retinal pathology. Future studies must determine how reversible these changes are after a hypothetical recovery of the damaged retina, and whether they affect the visual system’s functionality.

## Data availability statement

The raw data supporting the conclusions of this article will be made available by the authors, without undue reservation.

## Ethics statement

The animal study was reviewed and approved by the Comité Ético de Experimentación Animal de la Universidad de Castilla-La Mancha, Vicerrectorado de Investigación y Política Científica. Campus Universitario s/n 2071—Albacete.

## Author contributions

JM-G and EC: conception of the work and obtaining funding, data interpretation, and drafting the work. JM-G: design of experiments and quantitative analysis. All authors carried out experiments, revised the article critically for important intellectual content, accepted the final approval of the latest version to be published, and have made substantive contributions for having credit as authors.

## Footnotes

1
imagej.nih.gov/ij/

## Supplementary material

Supplementary Figure 1 Image processing to measure immunolabeling and percentage of immunoreactive area. **(A,B)** Example of original RGB images from VGLUT1 and VGLUT2 immunolabeling in the visual cortex of SD rats at P230. **(C,D)** The photomicrographs from upper panel converted to 8-bit grayscale images. **(E,F)** Applying the background subtraction (Fiji) on the images in panel **(C,D)**. Scale bar: 10 μm.

Supplementary Figure 2 Confocal images of the retinal vertical sections counterstained with DAPI. **(A,C)** The control retinas of Sprague Dawley rats (SD) showing the lamination of the normal retina. **(B,D)** Retinas from a P23H rhodopsin transgenic line 1 rat (P23H) showing thinning and loss of photoreceptor cells. ONL thickness reduced in the P23H retina **(B)** compared to the 30-day-old control SD **(A)**. At P230 **(D)**, the photoreceptor cell layer had disappeared in the P23H rats. GCL, ganglion cell layer; INL, inner nuclear layer; IPL, inner plexiform layer; is, inner segment of photoreceptors; NFL, nerve fiber layer; ONL, outer nuclear layer; OPL, outer plexiform layer; RPE, retinal pigment epithelium. Scale bar: 20 μm.

Supplementary Figure 3 Comparative brain size of the SD and P23H rats. At both studied ages, a notable decrease in the size of P23H in relation to the SD brains is observed. Scale bar: 1 cm.
