# Supplementary figures and images for: Pre- and postsynaptic alterations in the visual cortex of the P23H-1 retinal degeneration rat model
Source: Front Neuroanat. 2022 Oct 13;16:1000085. doi: 10.3389/fnana.2022.1000085 (PMC9608761; doi:10.3389/fnana.2022.1000085)

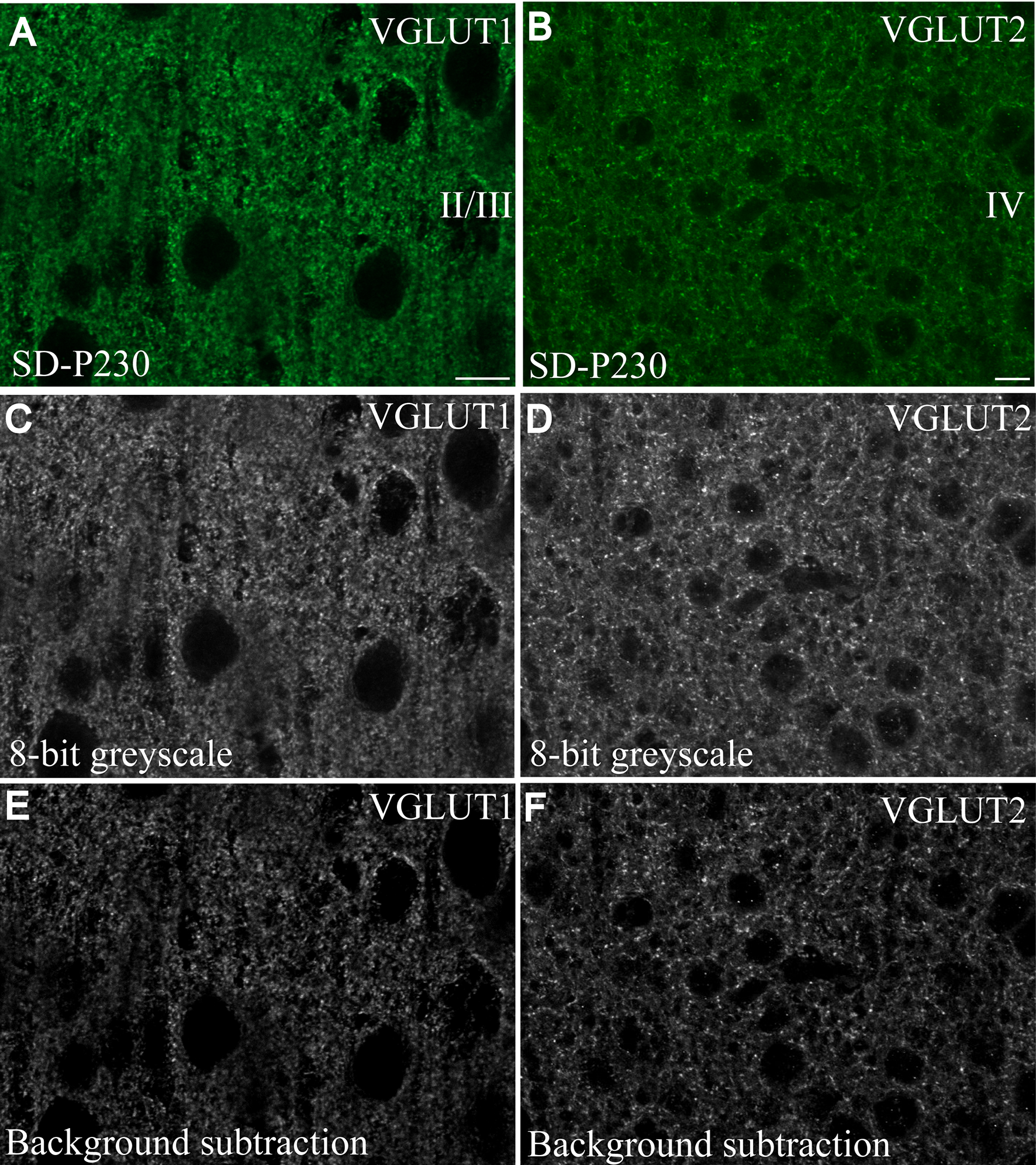

Supplement: Supplementary Figure 1 — Image processing to measure immunolabeling and percentage of immunoreactive area. (A,B) Example of original RGB images from VGLUT1 and VGLUT2 immunolabeling in the visual cortex of SD rats at P230. (C,D) The photomicrographs from upper panel converted to 8-bit grayscale images. (E,F) Applying the background subtraction (Fiji) on the images in panel (C,D). Scale bar: 10 μm. [file Image_1.TIF]

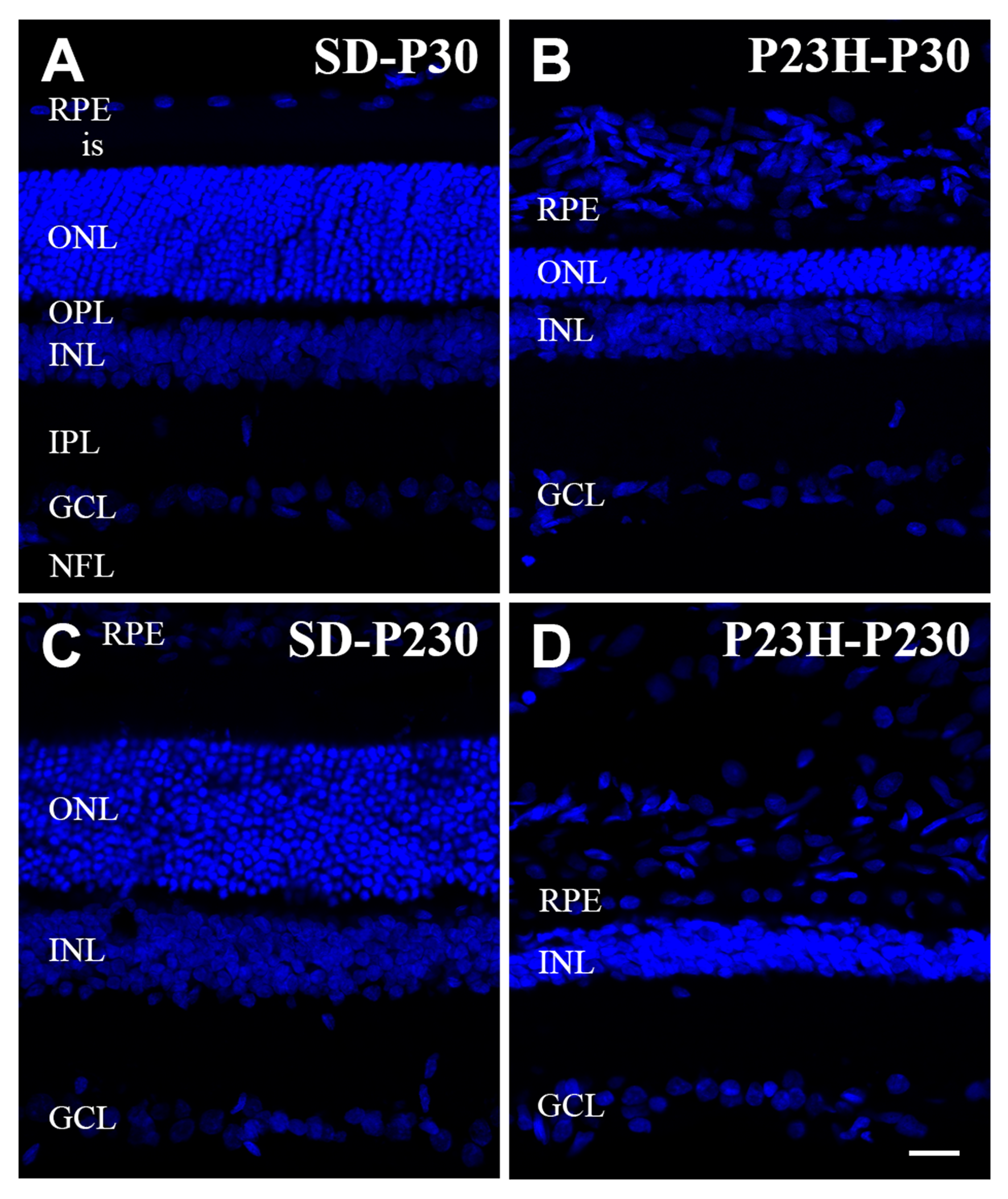

Supplement: Supplementary Figure 2 — Confocal images of the retinal vertical sections counterstained with DAPI. (A,C) The control retinas of Sprague Dawley rats (SD) showing the lamination of the normal retina. (B,D) Retinas from a P23H rhodopsin transgenic line 1 rat (P23H) showing thinning and loss of photoreceptor cells. ONL thickness reduced in the P23H retina (B) compared to the 30-day-old control SD (A). At P230 (D), the photoreceptor cell layer had disappeared in the P23H rats. GCL, ganglion cell layer; INL, inner nuclear layer; IPL, inner plexiform layer; is, inner segment of photoreceptors; NFL, nerve fiber layer; ONL, outer nuclear layer; OPL, outer plexiform layer; RPE, retinal pigment epithelium. Scale bar: 20 μm. [file Image_2.TIF]

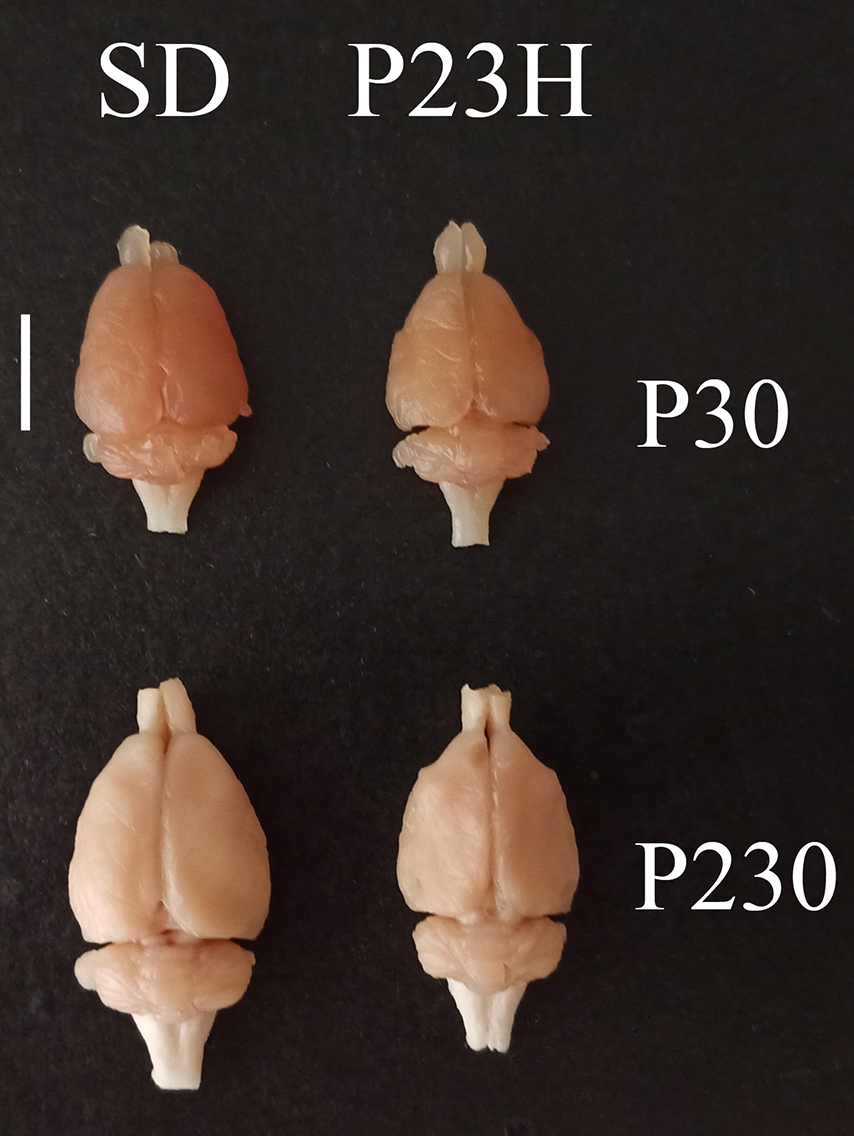

Supplement: Supplementary Figure 3 — Comparative brain size of the SD and P23H rats. At both studied ages, a notable decrease in the size of P23H in relation to the SD brains is observed. Scale bar: 1 cm. [file Image_3.TIF]
